# Accident vasculaire cérébral hémorragique mortel suite à une envenimation par une vipère à corne en Tunisie

**DOI:** 10.11604/pamj.2015.21.156.6401

**Published:** 2015-06-24

**Authors:** Hassen Ben Ghezala, Salah Snouda

**Affiliations:** 1 Faculté de Médecine de Tunis, Hôpital de Zaghouan, Avenue de l'Environnement, 1100 Zaghouan, Tunisie

**Keywords:** vipére, envenimations, AVC, hémorragie, CID, Viper, envenomation, stroke, hemorrhage, DIC

## Abstract

En Tunisie, parmi les envenimations par animaux terrestres, les morsures de vipéridés sont, après les piqûres de scorpions, les plus fréquentes. La présence d'un syndrome hémorragique est un des principaux critères de gravité. Nous rapportons le cas fatal d'une envenimation vipérine chez un patient de 37 ans sans antécédents qui a consulté nos urgences dans les suites d'une morsure de vipère. L'examen initial note un œdème local de la main gauche avec des ecchymoses et la trace de morsure. Initialement le patient est conscient. La biologie initiale est strictement normale. Quatre heures après son admission, le patient présente brutalement une altération de l’état de conscience. Le scanner cérébral pratiqué en urgence conclut à la présence d'hémorragie méningée, d'hématomes intra cérébraux et de lésions ischémiques diffuses. La biologie de contrôle révèle une coagulation intravasculaire disséminée (CIVD). L’évolution est rapidement défavorable avec décès du patient dans un tableau de coma dépassé.

## Introduction

En Tunisie, les envenimations vipérines occupent le deuxième rang après les morsures de scorpion. Les principales espèces identifiées existent surtout au centre et au Sud. La vipère à corne (*Cerastescerastes*) est l'une des espèces les plus dangereuses qui sévit surtout dans la région désertique de la Tunisie [1]. Les signes locaux sont dominés par le syndrome hémorragique, la nécrose et le syndrome des loges. Le syndrome hémorragique définit en général la gravité de ces envenimations et conditionne le pronostic vital. Nous rapportons le cas fatal d'une envenimation vipérine chez un patient de 37 ans.

## Patient et observation

**Figure 1:**
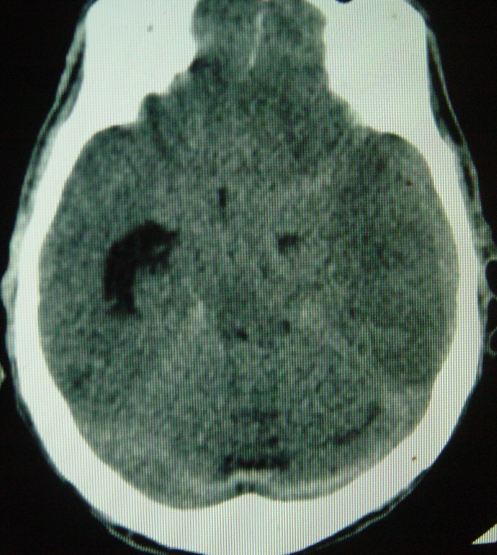
Hémorragie méningée avec des plages d'ischémie

**Figure 2:**
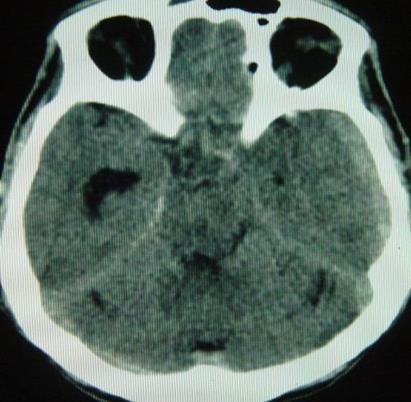
Engagement sous falcoriel

**Figure 3:**
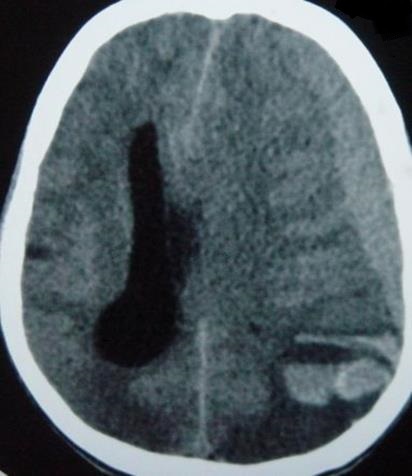
Hématome sous-dural

**Figure 4:**
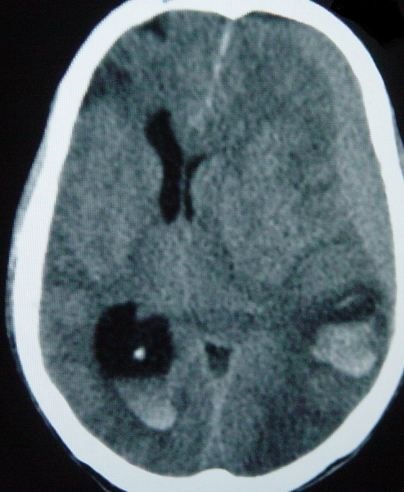
Hémorragie intra-ventriculaire

Il s'agit d'un patient de 37 ans sans antécédents qui a été transféré à nos urgences dans les suites d'une morsure de vipère. C'est un berger habitant la communauté de Nasrallah à 40 km de Kairouan dans le centre Ouest de la Tunisie qui a été mordu par une vipère de façon accidentelle au niveau de l'index de la main gauche. Sa famille déclare avoir trouvé sur place une vipère à corne. Il n'y a aucune notion ou de circonstance de traumatisme. A l ‘arrivée aux urgences: le patient présentait des vomissements et l'examen initial notait un œdème local de la main avec des ecchymoses et la trace de morsure. Initialement il était parfaitement conscient avec un état hémodynamique stable. La biologie initiale était strictement normale. Le patient a reçu immédiatement en 20 minutes une perfusion intraveineuse de sérum antivenimeux FAV-Afrique^®^ à la dose six ampoules associé à une expansion volémique par cristalloïdes, une antibiothérapie par amoxicilline-acide clavulanique, une analgésie par paracétamol et des soins locaux au niveau de la morsure. Il a reçu également de l'héparine calcique à doses prophylactiques. Il a été admis en salle d'accueil des urgences vitales. Quatre heures après l'admission, il présente brutalement une altération de l’état de conscience avec un score de Glasgow à 06/15, un signe de Babinski bilatéral, une anisocorie gauche et une rigidité de décérébration. Il devient hypotendu avec une PA à 70/35 mm Hg, des extrémités froides et des marbrures. Localement, il y a eu une extension de l’œdème à tout le membre supérieur gauche. Le bilan biologique pratiqué à 4 heures, montre une anémie aiguëavec une hémoglobine à 7.4 g/dl, une thrombopénie à 32000 éléments/mm^3^ et un TP effondré à 21%. Le reste du bilan d'hémostase trouve un fibrinogène inférieur à 0.5 et des produits de dégradation de fibrine (PDF) présents. La biologie à l'admission et après 4 heures est résumée dans le tableau 1 (Tableau 1). Le scanner cérébral sans injection pratiqué en urgence trouve une hémorragie méningée avec des plages d'ischémie (Figure 1), un engagement sous falcoriel (Figure 2), un hématome sous dural (Figure 3) et une hémorragie intra-ventriculaire (Figure 4). L’évolution est rapidement défavorable avec décès du patient dans un tableau de coma dépassé avec absence de tous les réflexes du tronc.

**Tableau 1:** Bilan biologique à l'admission et après 4 heures. La biochimie était peu perturbée

Bilan en hématologie	A l'admission	Après 4 heures
Hémoglobine (g/dl)	14	7,4
Globules blancs (/mm^3^)	16500	19600
Plaquettes (/mm^3^)	420000	32000
TP (%)	85	21 (INR à 3,24)
Fibrinogène (g/l)	3	<0,5
PDF, D-dimères	absents	présents

## Discussion

**Figure 5:**
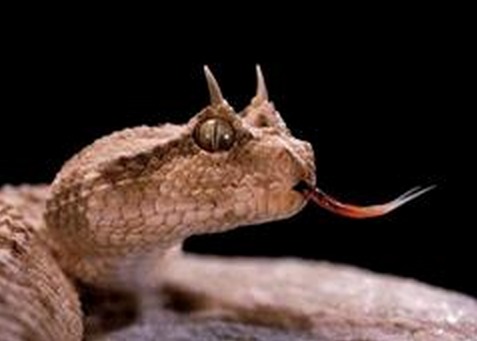
Vipère à corne: cerastescerastes

Les principales espèces de vipères identifiées en Tunisie sont la vipère lebetine (*Macroviperalebetina*) (du nord au sud), la vipère à corne (*Cerastescerastes*) (région désertique)(Figure 5), la vipère d'Avicenne (*Cerastesvipera*) (Gafsa et Tozeur) et la vipère des pyramides (*Echiscarinatuspyramidium*) (Médenine) [1]. Le venin de vipères est une salive qui est un mélange complexe d′enzymes protéiques et de toxines en quantités variables [2]. Sur le plan clinique, il existe des signes locaux avec une inflammation (constante), la trace de morsure (inconstante), des signes hémorragiques, une nécrose et un syndrome des loges. Il existe également des signes généraux avec un syndrome hémorragique et un état de choc pouvant être vagal, anaphylactique ou cardiogénique [3]. Les signes neurologiques sont rares et résultent souvent des troubles de l'hémostase. Le mécanisme de l'atteinte neurologique est complexe. L'atteinte neurologique est expliquée par une neurotoxicité directe par les neurotoxines alpha, béta et kappa. Les protéines: lectine C et désintégrine inhibent l'agrégation plaquettaire et la cascade de coagulation [4]. Dans le cas des vipères, et notamment de la vipère à cornes, la neurotoxicité est indirecte et induite par les lésions tissulaires et les perturbations de l′hémostase générées par les enzymes. Le venin de *Cerastescerastes* contient plusieurs protéines pro coagulantes: cérastocytine, et cérastobine. Il contient également la cérastase, la cérastatine, la cérastine et la cérastotine qui peuvent donner des thrombopénies et des hypofibrinogénémies [5]. Ces perturbations peuvent expliquer la survenue d'accidents vasculaires hémorragiques comme dans le cas de notre patient. Quelques cas rapportés dans la littérature ont décrit des coagulations intravasculaires disséminées (CIVD) secondaires à une envenimation par *Cerastescerastes* [6–8]. L'envenimation est classée en 4 grades (classification de Larréché et Goyffon) [9]. L'hospitalisation en réanimation est recommandée à partir du grade 2 et l'antivenin à partir aussi du grade 2. Le sérum administré dans cette observation n'est pas adapté puisqu'il n'est pas spécifique du genre *Cerastes*. L'antivenin adapté Favirept^®^ commercialisé en France par Sanofi Pasteur n'est pas disponible en Tunisie. D'où la nécessité absolu de disposer dans les services d'urgences d'Afrique du Nord des deux antivenins adaptés aux serpents d'Afrique du nord. Le traitement des complications neuro-vasculaires ne peut être dans ce cadre que préventif [10].

## Conclusion

L'envenimation vipérine, bien que rare, peut menacer le pronostic vital essentiellement par le biais du syndrome hémorragique systémique avec localisation viscérale neurologique comme le montre bien ce cas clinique.
